# The Distribution of Vascular Endothelial Growth Factor (VEGF), Human Beta-Defensin-2 (HBD-2), and Hepatocyte Growth Factor (HGF) in Intra-Abdominal Adhesions in Children under One Year of Age

**DOI:** 10.1155/2018/5953095

**Published:** 2018-12-30

**Authors:** Anna Junga, Māra Pilmane, Zane Ābola, Olafs Volrāts

**Affiliations:** ^1^Institute of Anatomy and Anthropology, Rīga Stradiņš University, Riga, Latvia; ^2^Department of Children Surgery, Rīga Stradiņš University, Riga, Latvia

## Abstract

The regulatory role between ischemia related factors and antimicrobial peptides in congenital intra-abdominal adhesions has not yet been defined. The aim of this research was to investigate the appearance and relative distribution of VEGF, HBD-2, and HGF in congenital intra-abdominal adhesions compared with relatively healthy tissue controls. The study group material was obtained from 48 patients who underwent abdominal surgery due to partial or complete bowel obstruction. VEGF, HBD-2, and HGF were detected using immunohistochemistry methods and their relative distribution was evaluated by means of the semiquantitative counting method. The results were analyzed using nonparametric statistic methods. A moderate number of VEGF positive endotheliocytes were detected, but there was no statistically significant difference between the groups. In the experimental group, a moderate to high number of VEGF positive macrophages was observed. In control group tissues, such macrophages were seen in significantly lower number (U = 61.0, p = 0.001). The increase of VEGF positive cells indicates support of angiogenesis due to the hypoxic conditions in case of adhesion disease. The number of HBD-2 marked fibroblasts and macrophages was moderate to high, but only few positive endotheliocytes were observed. Persisting appearance of HBD-2 positive structures might be a result of the inflammatory process. Most specimens showed occasional HGF positive macrophages and fibroblasts and there was no statistically significant difference between the groups. The relatively weak appearance of HGF suggests that the lack of this factor promotes the formation of fibrotic changes in case of intra-abdominal adhesions.

## 1. Introduction

Peritoneal adhesions are defined as pathological fibrotic bands that develop between any surfaces in the peritoneal cavity [1]. Depending on the etiology, peritoneal adhesions may be classified as congenital or acquired, of which the most frequent are postinflammatory or postoperative adhesions [2].

The pathogenesis of adhesion formation involves three important processes: (I) inhibition of the fibrinolytic and extracellular matrix degradation systems, (II) induction of an inflammatory response involving the production of cytokines and transforming growth factor-*β* (TGF-*β*), and (III) induction of tissue hypoxia, leading to increased expression of vascular endothelial growth factor (VEGF) [2]. Therefore, the aim was to research appearance of ischemia related factors and antimicrobial peptides in the tissue of congenital adhesions.

VEGF was first described by Senger et al. in 1983 as a tumor secreted “vascular permeability factor” [3]. It was originally identified as an endothelial cell specific growth factor stimulating angiogenesis [4]. In addition to endothelial cells, VEGF and VEGF receptors are expressed on numerous nonendothelial cells including macrophages, platelets, mesotheliocytes, and fibroblasts [5–7]. Macrophages play a key role in the induction of angiogenesis in fibroproliferative states [6]. Macrophage production of VEGF is increased under hypoxic conditions [5]. Fibroblasts may store VEGF in a latent state for urgent use in angiogenesis [8]. VEGF is upregulated in the postoperative adhesion formation [9].

Human beta-defensin-2 (HBD-2) could link inflammation and the host defense through its proangiogenic activity [10]. HBD-2 is cysteine-rich antimicrobial peptide of the human innate immune system with a broad antibacterial spectrum and rare bacterial resistance [11]. HBD-2 usually exhibits a stronger potential antimicrobial activity against gram-negative organisms [12]. HBD-2 may also enhance the inflammatory response since it is a specific chemoattractant for human neutrophils [13]. HBD-2 was originally described in psoriatic lesions and it is produced following stimulation of epithelial cells by contact with microorganisms or cytokines such as TNF-alpha and IL-1 [14]. HBD-2 is the main defensin present in the peritoneal membrane [15].

Hepatocyte growth factor (HGF), also named “hepatotropin,” is a heterodimer molecule composed of a 69 kDa alpha-subunit and a 34 kDa beta-subunit [16]. HGF is expressed in a wide variety of cells, including endothelial cells, macrophages, and fibroblasts [17, 18]. HGF targets vascular endothelial cells and is a potent angiogenic factor [19]. It stimulates proliferation and migration of various cell types via tyrosine phosphorylation of the HGF receptor, c-Met [20]. Mesothelial cell proliferation and migration play an important role in reducing formation of postoperative peritoneal adhesions [21]. HGF prevents peritoneal thickening and inhibits expression of TGF-*β*1 and type 1 collagen in rat peritoneum [22]. HGF strongly exerts intestinal adhesions by diminishing interferon-gamma (IFN-*γ*) production [23]. HGF has been reported to prevent the fibrosis within the peritoneum that is associated with enhanced peritoneal cell proliferation and viability [24].

The aim of this research was therefore to investigate the appearance and relative distribution of VEGF, HBD-2, and HGF in specimens of patients with congenital intra-abdominal adhesions.

## 2. Materials and Methods

The study was approved by the university ethics committee and was performed according to the principles of the Declaration of Helsinki.

The material was obtained from 48 patients (22 males, 26 females) who underwent abdominal surgery due to complete or partial bowel obstruction. 19 out of 48 specimens were evaluated as congenital adhesions (embryonic peritoneal adhesions, Ladd band), but 29 were evaluated as acquired adhesions related to gastrointestinal perforation, diffuse peritonitis, and repeated surgeries.

Most frequently adhesions were localized between the jejunal small intestinal loops and the proximal parts of ileum (20 cases) or at the duodenal region (ten cases). In four cases specimens were obtained from the distal parts of the ileum. In 12 cases adhesions were forming a Ladd band, but in another two cases the anterior abdominal wall was involved (see Table 1).

The control group was obtained from eight patients (six males, two females) with surgical repair of inguinal hernia (see Table 2). All patients were under one year of age.

The tissue material was fixed in Stefanini solution [25]. After fixation the study material was dehydrated, using alcohol solutions from 70° till 96°, and degreased in xylene solution. Then tissues were embedded in paraffin and the blocks of paraffinized tissues were sectioned into slides 3-4 *μ*m in thickness by means of a microtome (Leica RM2245, Leica Biosystems Richmond Inc., USA). To acquire an overview morphologic picture, the slides were processed for hematoxylin and eosin. Stained preparations were analyzed by light microscope (Leica DM500RB, Leica Biosystems Richmond Inc., USA).

## 3. Immunohistochemical Analysis

Tissue immunohistochemical staining for biomarkers identification was done by biotin-streptavidin method [26], using the following VEGF (code-orb191500, rabbit, polyclonal, working dilution 1:100, Biorbyt Ltd., United Kingdom), HBD-2 (code-sc-20798, rabbit, polyclonal, working dilution 1:100, Santa Cruz Biotechnology, Inc., USA), and HGF (code-AF-294-NA, goat, polyclonal, working dilution 1:300, R&D Systems, Germany) antibodies. All antibodies used in research were diluted with Antibody Diluent (code-938B-05, Cell Marque™, USA).

Adhesion tissue cuts were deparaffinized and washed in alcohol and water, then rinsed with TRIS buffer (code-2017X12508, Diapath S.p.A., Italy) twice for 5 minutes, and placed in boiling EDTA buffer (code-2017X02239, Diapath S.p.A., Italy) in microwave for 5 minutes. When the samples had cooled down, they were washed twice for 5 minutes in TRIS wash buffer. Further, blocking for 10 minutes in 3% peroxide solution was performed and then washed twice for 5 minutes in TRIS wash buffer. To decrease background staining, normal blocking serum for 20 minutes was used. All tissue samples were incubated with primary antibodies for 1 hour.

HiDef Detection™ HRP polymer system was used for the rabbit origin antibodies. The specimens were incubated for 10 minutes at room temperature with HiDef Detection™ Amplifier (code-954D-31, Cell Marque™, USA). Another washing for 5 minutes in TRIS wash buffer was performed. Further, incubation for 10 minutes at room temperature with HiDef Detection™ HRP Polymer Detector (code-954D-32, Cell Marque™, USA) was performed. After this processing, the preparations were rinsed for 5 minutes by TRIS buffer solution. After rinsing, DAB substrate-chromogen system (code-957D-60, Cell Marque™, USA) was used for 10 minutes to obtain positive structure staining in brown color.

For antibodies obtained from goat ImmunoCruz™ ABC staining system (code-sc-2023; Santa Cruz Biotechnology, Inc., USA) was used. After incubation of the specimens with the primary antibody for 1 hour at room temperature, rinsing three times for 5 minutes in TRIS buffer solution was performed. Tissue cuts were incubated for 30 minutes with biotin containing secondary antibody (biotinylated goat Ig). Then repeated sample rinsing for 5 minutes in TRIS buffer solution followed for three times and after that incubation with enzyme peroxidase bounded streptavidin for 30 min at room temperature. Then again washing in TRIS wash buffer for 5 minutes followed. Tissue coating with DAB substrate-chromogen system and incubation at room temperature, resulting in a positive structure coloring brown, was done for up to 10 minutes.

Regardless of the staining system, after incubation with chromogenic substrate system samples were rinsed in running water and counterstained with hematoxylin (code-05-M06002, Mayer's Hematoxylin, Bio Optica Milano S.p.A., Italy).

Finally, all samples were dehydrated in increasing concentration alcohols and clarified with carboxylic acid and xylene, as well as covering with the glue Pertex® (code-00811; HistoLab®, Sweden).

Slides were examined under the light microscope. Findings were photographed with a Leica DC 300F camera and analyzed with image-processing and analysis software Image Pro Plus.

The semiquantitative counting method was used for the registration of the relative amount of immunopositive structures [27]. The amount of structures was analyzed in five fields of view (magnification X 250) in a randomly selected section. The average amount of structures was chosen for further analysis. The designations were as follows:

  0: no positive structures in the visual field;
  0/+: occasionally positive structures in the visual field;
  +: few positive structures in the visual field;
  +/++: few to moderate positive structures in the visual field;
  ++: moderate positive structures in the visual field;
  ++/+++: moderate to numerous positive structures in the visual field;
  +++/++++: numerous to abundant positive structures in the visual field;
  ++++: abundant positive structures in the visual field.

## 4. Data Analysis

To characterize the research group descriptive statistic methods were used. For the description of each marker, median and interquartile range were used. Data analysis was conducted using nonparametric statistical methods. Spearman's rank correlation coefficient (rs) was calculated to evaluate correlation in between ischemia related factors and antimicrobial peptides. The acquired results were interpreted: rs ≤ 0.3, weak correlation; 0.3 < rs < 0.7, moderate correlation; rs ≥ 0.7, strong correlation. For the comparison of groups, Mann-Whitney U test was used. Two-tailed p values of <0.05 were considered as statistically significant. Statistical analysis was conducted using Statistical Package for the Social Sciences (SPSS) program version 23.0 (IBM Corp., Armonk, NY, USA).

## 5. Results

In the histologic overview specimen of the control group that was obtained during inguinal hernia repair, an overall normal histologic finding was seen. In all of the eight specimens, flat mesotheliocytes were found. In the submesothelial loose connective tissues, collagen fibers, fibroblasts, and rare, diffusely placed macrophages were seen. In two cases, a slight sclerosis of the blood vessel walls and in one of the specimens also angiogenesis without inflammatory changes were observed.

In the adhesion tissues round shaped mesotheliocytes and fibroblasts of modified shape were observed. Dense connective tissue bundles were placed chaotically and sometimes also large collagen fiber bundles without fibroblasts were seen. In almost all specimens, tissues were infiltrated by polymorphonuclear leukocytes and macrophages. In one part of the patient's specimen inflammatory cells were places diffusely, in the other part, perivascularly, often around sclerotized arterioles. In case of pronounced inflammation, epithelioid cells were seen. In the overview specimen angiogenesis and hyperemic vessels could be often seen.

**Figure 1:**
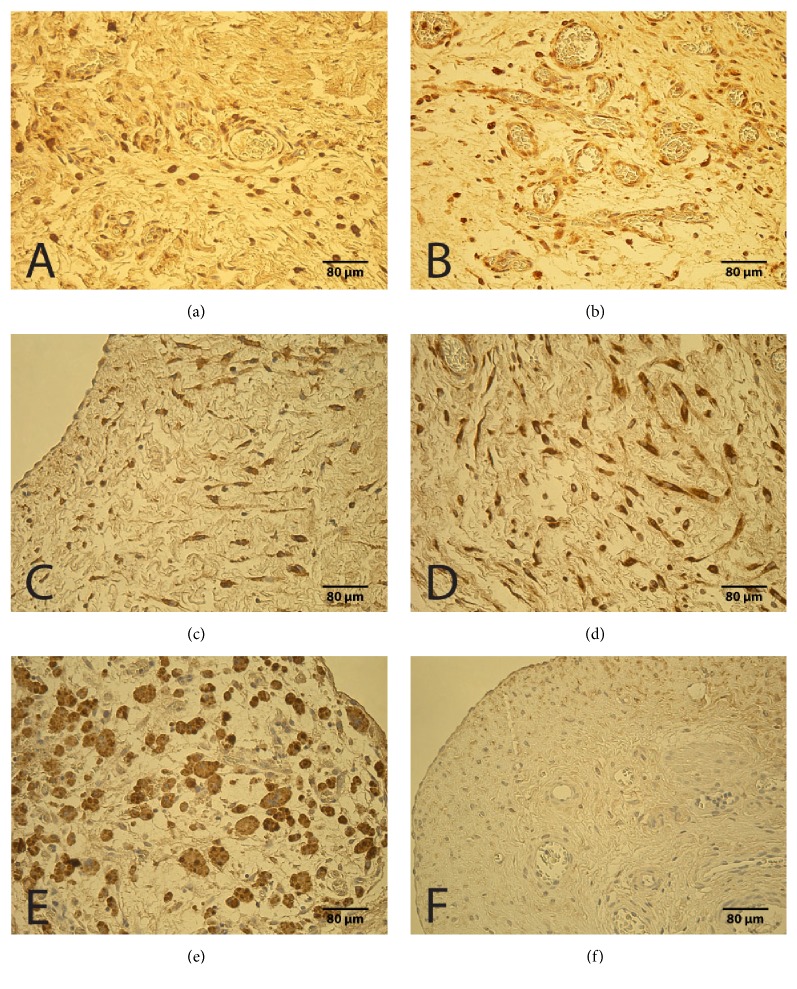
Immunoreactive structures in congenital adhesions and control group. (a) Moderate VEGF positive endotheliocytes, macrophages, and fibroblasts in congenital adhesions of a 48-day-old patient (VEGF IMH, x250). (b) Moderate VEGF positive endotheliocytes and fibroblasts of a 76-day-old patient in control group (VEGF IMH, x250). (c) Moderate HBD-2 positive fibroblasts, macrophages, and mesotheliocytes in congenital adhesions of a 151-day-old patient (HBD-2 IMH, x250). (d) Moderate HBD-2 positive fibroblasts, macrophages, and endotheliocytes of a 76-day-old patient in control group (HBD-2 IMH, x250). (e) Moderate HGF positive macrophages and epithelioid cells in congenital adhesions of a two-day-old patient (HGF IMH, x250). (f) Few HGF positive mesotheliocytes of a 53-day-old patient in control group (HGF IMH, x250).

In the experimental group, a moderate number of VEGF positive structures were detected, but there was no statistically significant difference between the groups in general (U = 124.0, p = 0.089). In 18 specimens, the number of marked structures was moderate to high (++/+++), in 20 specimens moderate (++), and in four specimens few to moderate (+/++). Only six cases showed few (+) or occasional (0/+) VEGF positive structures. A positive reaction for VEGF was found in endotheliocytes, mesotheliocytes, fibroblasts, and macrophages (Figure 1(a)). The control group tissues showed moderate (++) or few to moderate (+/++) appearance of VEGF positive cells (Figure 1(b)).

In the experimental group, moderate to high number of VEGF positive macrophages was observed. In control group tissues, such macrophages were seen in significantly lower number (U = 61.0, p = 0.001).

A positive reaction for HBD-2 was observed in fibroblasts, macrophages, endotheliocytes, and mesotheliocytes (Figure 1(c)). In 15 cases, the number of marked cells was high (+++), in 10 cases moderate to numerous (++/+++), and in seven cases moderate (++), but in nine cases few to moderate (+/++). Few (+) positive cells were found in six cases. In the control group tissues, HBD-2 positive structures (Figure 1(d)) were found in moderate (++) or moderate to high (++/+++) number and there was no statistically significant difference between the groups (U = 185.5, p = 0.951).

In the experimental group, only few positive endotheliocytes were observed. In control group tissues, positive endotheliocytes were seen in significantly higher counts for HBD-2 (U = 50.0, p < 0.001).

In the experimental group, HGF positive macrophages and fibroblasts were mostly seen in occasional (0/+) appearance. 16 specimens did not contain any HGF positive structures. Few (+) fibroblasts and macrophages contained this factor in ten specimens. Only in 5 cases few to moderate (+/++) or moderate (++) numbers of these structures were positive for HGF. A positive reaction for HGF was observed in epithelioid cells (Figure 1(e)). In the control group, most specimens also showed occasional HGF positive macrophages, fibroblasts, and mesotheliocytes (Figure 1(f)) and there was no statistically significant difference between the groups (U = 137.5, p = 0.182).

All semiquantitative results are summarized in Table 3.

Using Spearman's correlation test positive correlations were observed between the immunoreactive structures for HBD-2 and HGF (rs = 0.443, p = 0.002) and HBD-2 and VEGF (rs = 0.625, p < 0.001) as well as between HGF and VEGF (rs = 0.302, p = 0.037).

## 6. Discussion

Hypoxia, resulting from tissue injury, appears to play a role in the pathophysiology of wound healing and adhesion formation [28]. VEGF as a key mediator of angiogenesis is critical for vascular remodeling during tissue repair after inflammation or injury [29]. The mesothelial and vascular endothelial cells in the peritoneal blood vessels express VEGF [1]. Human peritoneal mesothelial cells contribute to the intraperitoneal production of VEGF, which may augment angiogenesis in the peritoneal membrane [7]. In addition to eliciting endothelial proliferation, VEGF also induces increased vascular permeability [30]. In our study the relatively low amount of VEGF positive endotheliocytes demonstrates that there was no pronounced hypoxia that could have stimulated neoangiogenesis.

However, there is data supporting that VEGF is upregulated in the process of adhesion development. It is considered a critical cytokine in the development of adhesions [9]. The upregulation of VEGF may be a compensatory mechanism regulating angiogenesis in order to provide nutrients and oxygen to the injured tissues [31]. Despite the lack of statistically significant difference between our study groups, predominantly moderate appearance of VEGF in the experimental group specimen suggests that production of VEGF may affect the development of congenital intra-abdominal adhesions. Our study reveals a more pronounced number of VEGF containing macrophages. Macrophage production of VEGF is increased under hypoxic conditions [5]. However, the causative link between hypoxia and angiogenesis in case of adhesion disease remains a speculation.

HBD-2 is an antimicrobial peptide upregulated during infection and inflammation [32]. The inducible antimicrobial peptide HBD-2 stimulated by proinflammatory cytokines and bacterial products is essential to antipathogen responses of gut epithelial cells [33]. In addition to their antimicrobial abilities, these peptides are potent mediators of inflammation with stimulatory effects on epithelial and inflammatory cells, influencing cell proliferation, cytokine/chemokine production, and chemotaxis. HBD-2 has been reported to be elevated in patients with chronic inflammatory conditions [34]. Thus, our data of moderate to numerous appearance of HBD-2 in congenital intra-abdominal adhesions might be the result of an inflammatory process.

HBD-2 might link inflammation and the host defense through its proangiogenic activity. HBD-2 stimulates chemotaxis of human endothelial cells with an extent similar to VEGF [10]. This might explain positive correlations between the immunoreactive structures for HBD-2 and VEGF.

HGF is a pleiotropic cytokine that may promote VEGF driven angiogenesis [35]. Induction of angiogenesis by HGF supplementation resulted in reduced local hypoxia. VEGF enhanced endothelial permeability and edema, whereas HGF inhibited endothelial permeability [20]. Combining VEGF with HGF can promote neovascularization [35]. Appearance of HGF and VEGD in intra-abdominal adhesions showed positive correlation and can be associated with vascular remodeling during adhesion formation.

HGF elicits the regression of peritoneal fibrosis, in which TGF-*β*-induced myofibroblasts are critical for tissue scarring [20]. HGF inhibits TGF-*β* production in cultures of myofibroblasts and antagonizes the actions of TGF-*β*, thus preventing fibrosis [36]. Moreover, HGF inhibits platelet derived growth factor overproliferation of myofibroblasts, reduces connective tissue growth factor induced scarring, and inhibits monocyte chemoattractant peptide-1 mediated inflammation during the HGF-mediated attenuation of fibrosis [20]. HGF strongly inhibits intestinal adhesion by diminishing IFN-*γ* production [23]. IFN-*γ* is a key molecule for abdominal adhesion formation after hepatectomy in mice, acting via the reciprocal balance between plasminogen activator inhibitor-1 and tissue plasminogen activator, the pivotal factors in fibrinolytic activity. HGF strongly inhibits adhesion formation by regulating IFN-*γ* and plasminogen activator inhibitor-1 [37]. Our study showed relative weak appearance of HGF. Therefore, the lack of this factor might support formation of congenital intra-abdominal adhesions.

## 7. Conclusions

The increase of VEGF positive cells indicates support of angiogenesis due to the hypoxic conditions in case of adhesion disease.

Persisting appearance of HBD-2 positive structures might be a result of the inflammatory process.

The relatively weak appearance of HGF suggests this as possible fibrosis supporting factor in the pathogenesis of intra-abdominal adhesions.

VEGF, HBD-2, and HGF are part of the repair mechanisms in the peritoneum.

## Figures and Tables

**Table 1:** Experimental group.

	Age (days)/ Sex	Location		Age (days)/ Sex	Location		Age (days)/ Sex	Location
1	0/M	SI	17	4/F	SI	33	56/M	SI
2	0/F	D	18	9/F	D	34	56/F	SI
3	0/F	LB	19	9/F	D	35	62/F	D
4	1/M	SI	20	9/F	AAW	36	67/M	SI
5	1/F	LB	21	9/M	LB	37	71/F	SI
6	1/M	SI	22	14/M	SI	38	94/F	SI
7	1/F	LB	23	15/M	LB	39	100/F	SI
8	1/F	D	24	19/F	SI	40	103/M	D
9	1/M	AAW	25	26/F	LB	41	108/M	DI
10	2/M	SI	26	28/M	DI	42	129/M	DI
11	2/M	SI	27	30/F	SI	43	130/F	D
12	2/M	D	28	36/F	SI	44	134/F	SI
13	2/M	LB	29	39M	D	45	151/F	SI
14	3/M	LB	30	41/F	LB	46	185/F	SI
15	4/M	D	31	48/M	LB	47	210/M	DI
16	4/F	LB	32	51/F	LB	48	292/F	SI

Abbreviations: M, male; F, female; SI, small intestine; DI, distal ileum; D, duodenum; LB, Ladd's band; AAW, anterior abdominal wall.

**Table 2:** Control group.

	Age (days)/Sex
1	46/F
2	53/M
3	56/M
4	60/F
5	73/M
6	76/M
7	92/M
8	145/M

Abbreviations: M, male; F, female.

**Table 3:** Semiquantitative evaluation of immunoreactive structures.

		Experimental grouptb	Control group	U	p
VEGF	Median	++	++	124.0	0.089
	IQR	0.5	0.5		
HBD-2	Median	++/+++	++/+++	185.5	0.951
	IQR	1.5	0.5		
HGF	Median	0/+	0/+ to +	137.5	0.182
	IQR	1.0	0.85		

Abbreviations: VEGF, vascular endothelial growth factor; HBD-2, human beta-defensin-2; HGF, hepatocyte growth factor; IQR, interquartile range; U, Mann Whitney U value. Quantification of structures: 0, no positive structures in the visual field; 0/+, occasionally positive structures in the visual field; +, few positive structures in the visual field; +/++, few to moderate positive structures in the visual field; ++, moderate positive structures in the visual field; ++/+++, moderate to numerous positive structures in the visual field.

## Data Availability

Patient data used to support the findings of this study are restricted by the Rīga Stradiņš University ethics committee in order to protect patient privacy. Immunohistochemical data are available from the corresponding author upon request.
